# Current and future advances in practice: IgG4-related disease

**DOI:** 10.1093/rap/rkae020

**Published:** 2024-04-10

**Authors:** Zachary S Wallace, Guy Katz, Yasmin G Hernandez-Barco, Matthew C Baker

**Affiliations:** Division of Rheumatology, Allergy, and Immunology, Massachusetts General Hospital, Boston, MA, USA; Harvard Medical School, Harvard University, Boston, MA, USA; Division of Rheumatology, Allergy, and Immunology, Massachusetts General Hospital, Boston, MA, USA; Harvard Medical School, Harvard University, Boston, MA, USA; Harvard Medical School, Harvard University, Boston, MA, USA; Division of Gastroenterology, Massachusetts General Hospital, Boston, MA, USA; Division of Immunology and Rheumatology, Stanford University, Palo Alto, CA, USA

**Keywords:** IgG4-related disease, epidemiology, outcomes, treatment, rituximab, glucocorticoids

## Abstract

IgG4-related disease (IgG4-RD) is an increasingly recognized cause of fibroinflammatory lesions in patients of diverse racial and ethnic backgrounds and is associated with an increased risk of death. The aetiology of IgG4-RD is incompletely understood, but evidence to date suggests that B and T cells are important players in pathogenesis, both of which are key targets of ongoing drug development programmes. The diagnosis of IgG4-RD requires clinicopathological correlation because there is no highly specific or sensitive test. Glucocorticoids are highly effective, but their use is limited by toxicity, highlighting the need for studies investigating the efficacy of glucocorticoid-sparing agents. B cell-targeted therapies, particularly rituximab, have demonstrated benefit, but no randomized clinical trials have evaluated their efficacy. If untreated or under-treated, IgG4-RD can cause irreversible organ damage, hence close monitoring and consideration for long-term immunosuppression is warranted in certain cases.

Key messages

- IgG4-related disease (IgG4-RD) can affect patients of diverse racial and ethnic backgrounds and can lead to irreversible damage if not treated.
- Nearly any organ can be affected by IgG4-RD, but common sites include the salivary glands, lacrimal glands, orbit, pancreatobiliary system, lung, kidney and retroperitoneum.
- B and T cells are thought to be important in the pathogenesis, whereas the IgG4 molecule is often not considered a driver of disease.
- The diagnosis requires clinicopathological correlation because there are no highly sensitive or specific tests, including serum IgG4 concentrations and IgG4^+^ plasma cell infiltrates.
- Glucocorticoids are highly effective for IgG4-RD but are associated with toxicities, hence CS-sparing drugs are now being investigated as treatments.

## Introduction

The condition that would become known as IgG4-related disease (IgG4-RD) was first described in 2001 [1]. Since this description of 20 patients in Japan with autoimmune pancreatitis (AIP) and elevated serum IgG4 concentrations, diverse organ involvement with and without elevated IgG4 levels has been described in cohorts worldwide. Prior to 2001, this was a disease known by various names, mostly eponyms, depending on the manifestation: Kuttner’s tumour (submandibular sialoadenitis), Riedel’s thyroiditis, Ormond’s disease (retroperitoneal fibrosis), Mikulicz syndrome (symmetric lacrimal and salivary gland disease) and AIP. These diverse manifestations share similar histopathological and immunohistochemical findings [2]. Recent advances have informed our understanding of the epidemiology of IgG4-RD, its pathogenesis, approaches to diagnosis and effective treatments.

## Epidemiology of IgG4-RD

IgG4-RD tends to affect people in their fifth to seventh decades of life, but paediatric and older adult patients can also present with IgG4-RD. Most cohorts demonstrate a male predominance; however, this varies: pancreatobiliary disease and retroperitoneal disease more commonly affect males, whereas disease limited to the head and neck most commonly affects females. Population-based estimates of IgG4-RD incidence and prevalence are limited. Using a claims-based algorithm to identify cases in the USA, the estimated incidence was 0.78–1.39 per 100 000 person-years between 2015 and 2019, and the point prevalence as of 1 January 2019 was 5.3 per 100 000 persons [3, 4]. The incidence of pancreatic disease in Japan was estimated to be higher (3.1 per 100 000 persons) using different methods [5]. Patients with IgG4-RD may have an elevated risk of death compared with the general population [6], probably driven, in part, by irreversible organ damage from IgG4-RD in addition to treatment complications; the precise cause of excess mortality is unknown.

## Patterns of presentation

The diverse manifestations of IgG4-RD can make the diagnosis difficult to establish [7], hence a high level of suspicion for IgG4-RD is needed to avoid diagnostic delays. Although nearly any organ can be affected, four typical presentations are most common (Table 1): head and neck disease, systemic disease, hepato-pancreatobiliary disease and retroperitoneal fibrosis/aortic disease [8].

**Table 1.** Patterns of presentation

Pattern	Pancreato-hepatobiliary disease	Retroperitoneum and aorta	Head- and neck-limited disease	Mikulicz and systemic disease
Typical manifestations	Autoimmune pancreatitis, sclerosing cholangitis	Retroperitoneal fibrosis, aortitis, large vessel disease	Salivary and/or lacrimal gland enlargement, adnexal orbital involvement	Classic symmetric lacrimal, salivary gland enlargement with involvement in the chest and/or abdomen
Male predominance	Yes	Yes	No	Yes
Age, mean, years	63	58	55	63
Serum IgG4 concentration	Elevated	Normal to mildly elevated	Elevated	Very high
Examples of potential mimics	Pancreatic cancer, autoimmune pancreatitis type 2, primary sclerosing cholangitis	Lymphoma, Erdheim–Chester disease, GCA	SS and other autoimmune CTD, granulomatosis with polyangiitis, lymphoma	

Adapted from Wallace ZS, *et al.*, Clinical phenotypes of IgG4-related disease: an analysis of two international cross-sectional cohorts. *Ann Rheum Dis* 2019; 78:406.

### Head and neck disease with or without systemic involvement

Disease in the head and neck is often appreciable on physical examination. The most common manifestations include salivary gland and/or lacrimal gland enlargement, lymphadenopathy and orbital disease (e.g. orbital myositis, orbital pseudotumour). Salivary or lacrimal gland disease typically presents with painless swelling, often symmetrically. Orbital involvement often causes proptosis; it can cause pain and change in vision owing to involvement of extra-ocular muscles and/or optic nerve compression. Less common manifestations include pachymeningitis, thyroiditis and hypophysitis. Disease isolated to the head and neck tends to occur more often in females. Head and neck involvement in combination with systemic disease (e.g. pancreas, biliary tract, kidneys, where tubulointerstitial nephritis can occur) is associated with high serum IgG4 concentrations, frequent elevations in acute phase reactants and/or hypocomplementaemia. Many patients, especially those with head and neck involvement, have atopic disease (e.g. seasonal allergies) [9]; however, the significance of atopic disease in IgG4-RD in general is poorly understood. The symptoms of seasonal allergies are often distinct from IgG4-RD, except for sinusitis, which can be present in both seasonal allergic conditions and IgG4-RD. However, sinusitis from IgG4-RD is not seasonal and can lead to damage.

### Retroperitoneum and large vessel involvement: a fibrotic phenotype

Some manifestations tend to present with a fibrotic phenotype, of which retroperitoneal fibrosis is prototypic. Biopsies of these lesions demonstrate prominent fibrosis and less inflammation. Retroperitoneal fibrosis typically presents as soft tissue surrounding the infrarenal aorta, extending distally to involve the iliac arteries and often laterally to encase and medialize the ureters. Patients can have groin or back pain but are often asymptomatic and present with renal failure from ureteral obstruction and hydronephrosis. Other fibrotic manifestations include orbital pseudotumours, thyroiditis and sclerosing mesenteritis and mediastinitis. Patients with these manifestations in isolation frequently have normal serum IgG4 concentrations. The diagnosis is often difficult to establish, especially when a biopsy reveals few features other than fibrosis.

### Pancreatobiliary disease

Type 1 AIP (lymphoplasmacytic sclerosing pancreatitis) is one of the most common manifestations of IgG4-RD [10]. Of the patients with IgG4-related pancreatic disease, ≤20% also have biliary involvement manifesting as IgG4-related sclerosing cholangitis or biliary compression from pancreatitis. The most common presentation is painless jaundice, occurring in 70% of patients [11–14]. Imaging in AIP can present as diffuse or focal involvement (reviewed below). Less commonly, patients can present with acute pancreatitis. Type 2 AIP (idiopathic duct centric pancreatitis) is associated with IBD, not IgG4-RD, and will not be discussed here (Supplementary Table S1, available at *Rheumatology Advances in Practice* online) [15, 16].

## Current understanding of pathogenesis

**Figure 1.**
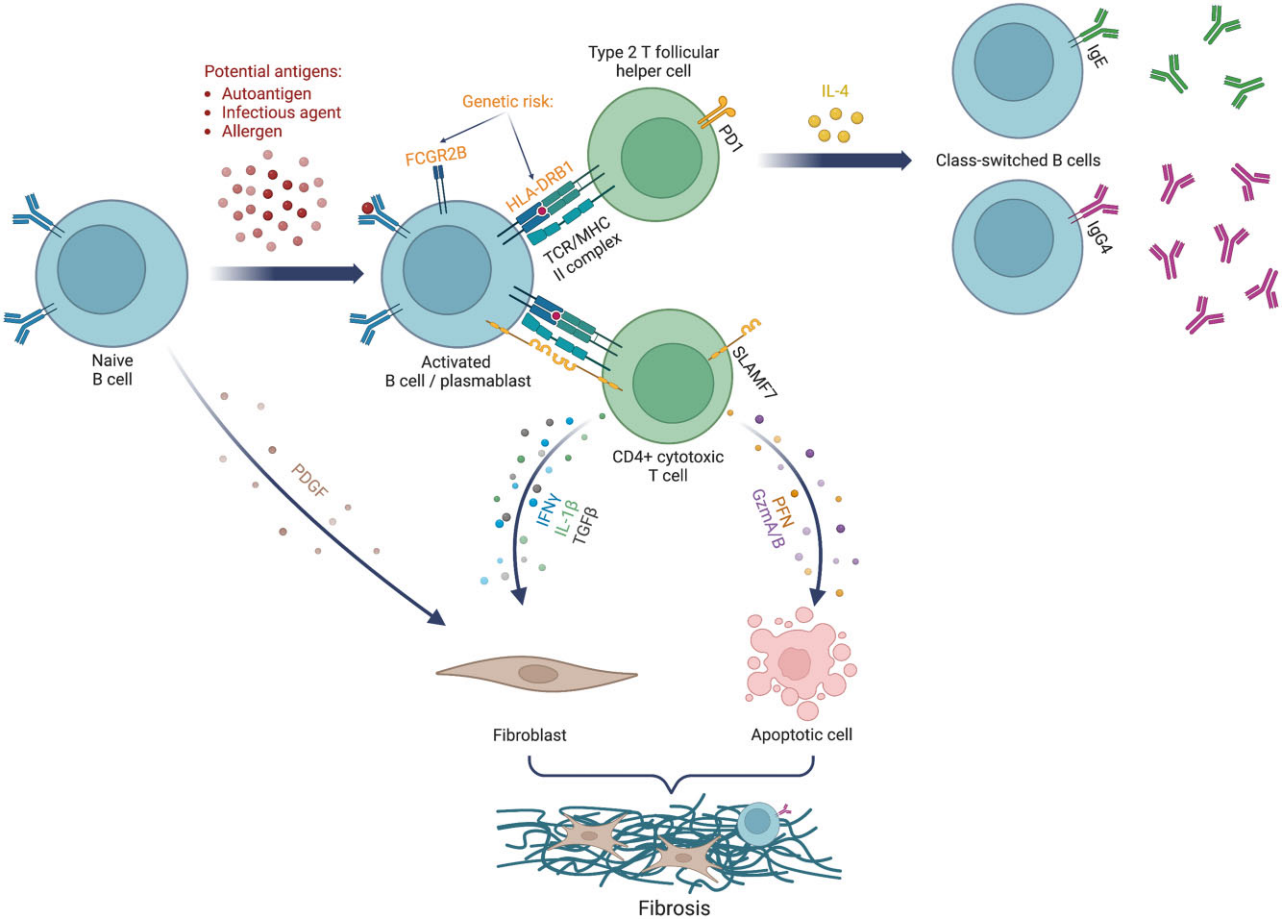
IgG4-related disease pathogenesis

The aetiology of IgG4-RD and the role that IgG4 plays in its pathogenesis remain poorly understood. Environmental exposures and genetic factors might place certain individuals at an increased risk for IgG4-RD. Dysregulated immunity, characterized by an expansion of CD4^+^ cytotoxic T lymphocytes, is a key hallmark of the disease (Fig. 1) [15–17].

### The role of the IgG4 molecule

Although not specific, most patients with IgG4-RD have an elevated serum IgG4 concentration. The degree of elevation is correlated with organ involvement and risk of relapse [18–20]. It remains unknown whether IgG4 is directly involved in pathogenesis, is a compensatory response to immune activation or is simply an epiphenomenon related to a misdirected immune response. IgG4 is considered an anti-inflammatory immunoglobulin because it undergoes Fab-arm exchange, which limits its ability to cross-link antigen effectively, it weakly fixes complement and it has a reduced capacity to bind activating Fc receptors but a preserved ability to bind inhibitory Fc receptors [21–23]. However, antigen-specific IgG4 antibodies can be pathogenic in certain conditions, as seen in muscle-specific tyrosine kinase myasthenia gravis, pemphigus foliaceus, pemphigus vulgaris, primary membranous nephropathy and chronic inflammatory demyelinated polyradiculoneuropathy [24].

### The role of B cells

Several observations support the central role of B cells in IgG4-RD. First, the pathological hallmarks include a lymphoplasmacytic infiltrate rich in IgG4^+^ plasma cells, in addition to storiform fibrosis with or without obliterative phlebitis [2]. Second, elevations in serum immunoglobulin concentrations, including IgG4, are common [25, 26]. Third, patients have oligoclonally expanded plasmablasts, which decrease in remission [27]. Fourth, the resurgence of plasmablasts and memory B cells is correlated with an increase in disease activity [27, 28]. Despite these observations, the precise role of B cells remains uncertain and is an active area of investigation [29, 30].

### The role of T cells

Two T cell subsets are thought to play key roles in IgG4-RD. Circulating type 2 T follicular helper cells expressing programmed cell death protein 1 are expanded in IgG4-RD. Their frequency is correlated with the number of organs involved, number of plasmablasts, IgG4 concentrations and IL-4 concentrations [30–32]. . Circulating type 2 T follicular helper cells produce IL-4, which is involved in class-switching of B cells to both IgG4 and IgE. T follicular helper cells expressing IL-4 are also expanded in affected tissue, where they promote B cell isotype switching, affinity maturation and oligoclonal expansion of IgG4^+^ B cells [33].

CD4^+^ effector memory T cells (defined as CD27^−^ CD62L^−^) expressing SLAMF7 are also expanded in IgG4-RD and found in affected tissue, and they decline in number with treatment [17]. Also known as CD4^+^ cytotoxic T lymphocytes, these cells express perforin, granzymes, granulysin and other mediators of cytotoxicity and produce profibrotic cytokines (e.g. TGF-β, IFN-γ and IL-1β) [17]. They may also be involved in apoptosis.

### Knowledge gaps in pathogenesis

The clonal expansion of both plasmablasts and CD4^+^ cytotoxic T lymphocytes seen in patients with IgG4-RD suggests that there might be a common antigen driving the disease, perhaps an autoantigen [27]. Proposed, but unconfirmed, potential autoantigens include carbonic anhydrase, plasminogen binding protein, lactoferrin, pancreatic secretory trypsin inhibitor, amylase alpha-2A, trypsinogen, annexin A11, laminin-511-E8, galectin-3 and IL-1 receptor antagonist [34–44]. The genetic contribution to IgG4-RD is also unclear. One genome-wide association study [45] found HLA-DRB1 and FCGR2B regions as susceptibility loci for IgG4-RD. Future work should continue to focus on autoantigen discovery, correlation of autoantibodies with specific organ involvement, the development of an animal model that recapitulates human disease and the identification of genetic risk factors. Understanding the inciting event that leads to disease onset and whether T cells and B cells are responding to the same antigen are key knowledge gaps that need to be addressed.

## Approach to the diagnosis of IgG4-RD

### Clinical practice

Although not meant for establishing a clinical diagnosis of IgG4-RD, the 2019 ACR/EULAR Classification Criteria for IgG4-RD provide a useful framework for evaluating a patient who might have IgG4-RD [46]. IgG4-RD should be suspected when a patient presents with a mass lesion (e.g. pancreatic mass or salivary gland enlargement) or wall thickening (e.g. biliary tract or aorta) in a characteristic organ, which includes the pancreas, salivary glands, bile ducts, orbits, kidneys, lungs, aorta, retroperitoneum, pachymeninges and/or thyroid gland [46]. Although nearly any organ can be affected, certain locations, such as the gut lumen, brain and bones, would be unusual. In most cases, the diagnosis is confirmed or supported by biopsy; however, this is not always possible because of the lesion location, procedure risk or patient preference. Regardless of biopsy findings, the diagnosis is established by clinicopathological correlation; no single finding by examination, pathology, imaging or laboratory tests is diagnostic. In classic presentations, a diagnosis can be made without a biopsy, assuming that mimicking conditions have been exonerated.

Imaging and laboratory tests are often useful when evaluating for IgG4-RD. In certain scenarios, classic radiographic findings (e.g. diffuse pancreatic enlargement with loss of lobulations and a halo sign around the pancreas, seen in 40% of patients with AIP) lend strong support to the diagnosis [47]. In other situations, such as a pancreatic mass, the diagnosis is challenging, and malignancy must be excluded. An elevated serum IgG4 concentration, especially at high levels, can support the diagnosis. However, IgG4 concentrations are neither highly sensitive nor specific to IgG4-RD, and elevations have been reported in pancreatic adenocarcinoma cases [25]. Additional laboratory findings, such as eosinophilia, elevated IgE, hypergammaglobulinemia and hypocomplementaemia can be observed in IgG4-RD [48–51].

Some laboratory tests can help to exclude IgG4-RD. Acute phase reactants, such as ESR and CRP, can be elevated but usually mildly so, particularly CRP; if either is very high (e.g. greater than several times the upper limit of normal), alternative diagnoses are likely. In addition, common mimickers of IgG4-RD, such as SS and ANCA-associated vasculitis, should be evaluated with anti-Ro, anti-La, anti-MPO and anti-PR3 antibodies, which are typically absent in IgG4-RD.

When evaluating for IgG4-RD, a physical examination should be conducted to evaluate for common manifestations, such as lacrimal and/or salivary gland enlargement. Cross-sectional imaging of the chest, abdomen and pelvis is recommended to assess for other manifestations that might support the diagnosis and be amenable to biopsy. CT, MRI and fluoro-deoxyglucose PET can be useful in the work-up [52–56].

### Histology of IgG4-RD

Tissue biopsy remains helpful in many cases to support a diagnosis of IgG4-RD and to rule out alternative diagnoses. Characteristic pathological features of IgG4-RD include a dense lymphoplasmacytic infiltrate rich in IgG4^+^ plasma cells and CD4^+^ T cells [2]. This infiltrate is often accompanied by fibrosis that has a storiform pattern. The word storiform derives from the Latin word *storea*, or woven mat, describing the irregular, whorled pattern of fibrosis observed. Obliterative phlebitis, a destruction of venous walls and obstruction of their lumen with immune cell infiltration and collagen deposition, might be observed. Obliterative arteritis is less commonly seen. These features, however, are distinct from necrotizing vasculitis; indeed, necrosis, microabscesses and prominent neutrophilic infiltrates are not expected in IgG4-RD.

IgG4^+^ plasma cells are increased in affected tissue. Although an infiltrate of ≥50 IgG4^+^ plasma cells per high-power field and/or an IgG4^+^:IgG^+^ plasma cell ratio of >40% strongly support the diagnosis, there are no universally accepted cut-offs. The histological and immunohistochemical findings (e.g. IgG4^+^:IgG^+^ cut-offs) might vary across organs affected by IgG4-RD. For instance, lacrimal gland biopsies might have less storiform fibrosis than biopsies from other organs [57, 58]. Of note, it is challenging to diagnosis IgG4-RD from a lymph node biopsy because, in part, the presence of IgG4^+^ plasma cells in lymph nodes is not considered specific to IgG4-RD [46, 59, 60].

### Considerations for diagnosing IgG4-related pancreatic disease

In addition to the 2019 ACR/EULAR IgG4-RD Classification Criteria, patients with pancreatic disease can also be evaluated for IgG4-RD using the HISORt (histology, imaging, serology, other organ involvement, response to therapy) criteria [61]. AIP classically presents with either diffuse or focal involvement, and certain pancreatic imaging findings can be supportive of a diagnosis of IgG4-RD [47, 62]. Diffuse involvement will often have more classic imaging features, including diffuse enlargement with loss of lobulations, characteristics of a sausage-shaped pancreas, long pancreatic duct strictures traversing more than one-third of the pancreas without downstream dilatation, a hyperenhancing thin rim surrounding the pancreas (also known as the halo sign) and hyperenhancement on venous phase. Focal involvement usually presents as a mass in the head of the pancreas leading to common bile duct dilatation (see IgG4-RD sclerosing cholangitis below). Focal AIP does not typically cause compression and subsequent dilatation of the main pancreatic duct. Pancreatic duct dilatation or vascular involvement should prompt evaluation for adenocarcinoma.

There are no guidelines regarding when a pancreatic biopsy should be pursued, but exclusion of malignancy is required in all focal AIP via endoscopic US with fine needle biopsy to obtain a core biopsy [63, 64]. Although fine needle aspiration can establish the diagnosis of malignancy, it is insufficient to establish a diagnosis of AIP because sample architecture is lost.

### Pitfalls of diagnosis

Establishing a diagnosis can be challenging when a biopsy is not feasible. Biopsy confirmation is especially important if there are unusual manifestations or other aspects of the history suggesting an alternative diagnosis. In general, one must not anchor on the sole finding of IgG4^+^ plasma cells in tissue to establish the diagnosis, because it is not a specific finding [59].

The serum IgG4 concentration is an important component when assessing IgG4-RD, but it is also not specific for IgG4-RD [65, 66]. Among patients with serum IgG4 testing in a health-care system, the positive predictive value of a level >135 mg/dl was 34% [25]. Among patients with pancreatic disease, the positive predictive value of a level >140 mg/dl was 36% [67]. Even when very high (more than five times the upper limit of normal), the positive predictive value was 73%; thus 27% of patients with an IgG4 concentration more than five times the upper limit of normal had an alternative diagnosis [68]. These data demonstrate the importance of considering a broad differential when evaluating patients with an elevated serum IgG4 concentration.

## Treatment

The goal of treatment in IgG4-RD is to reduce disease activity and prevent irreversible damage. Without treatment, the natural history in some is to accrue new organ involvement over time; therefore, patients who are untreated (e.g. mild salivary gland disease or resected disease from a single site) should be monitored closely. The response to treatment can vary based on the organ(s) involved and the duration of disease, both of which can be associated with the amount or stage of fibrosis; ultimately, the degree of fibrosis can dictate responsiveness to current treatments. The treatment goal for most patients is to induce and then maintain remission, but this approach should be personalized according to individual patient characteristics, disease manifestations and preferences. When evaluating disease activity, it is important to consider whether a manifestation is highly fibrotic and unlikely to respond to therapy (i.e. has significant damage).

### Inducing remission

Remission is the state in which the disease manifestations have either resolved or returned to a newly established baseline and is assessed using evaluations tailored to the specific organs involved. Although organs can have damage and fibrosis that is irreversible, most patients have significant reduction in the size of lesions and improvement in laboratory parameters with appropriate treatment.

Worldwide, glucocorticoids are first-line therapy for IgG4-RD [69]. The usual initial dose is 0.5–1.0 mg/kg of prednisone, based on the severity of the presentation. The optimal glucocorticoid regimen is unknown, but the initial dose is typically continued for 2–4 weeks and then tapered off over 2–3 months. Although effective, glucocorticoids have many toxicities (e.g. diabetes), especially in this older population that often has pancreatic damage and other co-morbidities [70]. Furthermore, glucocorticoids rarely provide a durable treatment response once stopped, and most patients flare within 3 years of glucocorticoid discontinuation [71].

Given the toxicities and brief response, conventional DMARDs are often combined with glucocorticoids for induction therapy. The decision to use a DMARD upfront is often guided by the patient’s manifestations (e.g. risk for damage with relapse), demographics and co-morbidities (e.g. high risk for CS toxicity), risk of future flare (e.g. very high serum IgG4 at baseline, multi-organ disease) and patient preference. Prospective studies (Table 2) have compared glucocorticoid monotherapy *vs* glucocorticoids plus either MMF, CYC or LEF [75–77]. In all cases, combination therapy improved both remission and relapse rates compared with glucocorticoids alone. Retrospective studies and case series have also reported benefit with other treatments, including MTX, AZA and iguratimod [84–86]. No studies have evaluated the comparative efficacy of CS-sparing agents. Larger randomized controlled trials of CS-sparing therapies are needed to understand their role.

**Table 2.** Clinical trials and comparative effectiveness studies in IgG4-related disease

Study/trial	**Study design** ^a^	Study arms	Primary outcome	Follow-up time	Results/status
**Glucocorticoids**					
Masaki *et al.* (2017) [72]	Single-arm open-label trial	PSL 0.6 mg/kg/day tapered to maintenance ≤10 mg (median 7 mg/day), *n* = 61	CR	12 months	62% complete remission 5% ultimately determined not to have IgG4-RD 29 of 44 (66%) with definite IgG4-RD had complete remission at 1 year
Wu *et al.* (2017) [73]	Open-label RCT	Group 1: high-dose prednisone: 0.8–1.0 mg/kg/day tapered to maintenance 7.5–10 mg/day, *n* = 21 (1 w/d) Group 2: medium-dose prednisone: 0.5–0.6 mg/kg/day tapered to maintenance 7.5–10 mg/day, *n* = 20 (1 w/d)	CR	24 months	CR at 12 weeks: 95% (group 1) *vs* 95% (group 2), *P* = 1.00 CR at 24 weeks: 95% (group 1) *vs* 80% (group 2), *P* = 0.157
Masamune *et al.* (2017) [74]	Open-label RCT (AIP)	Group 1: PSL 0.6 mg/kg/day tapered over 12 weeks to maintenance 5–10 mg/day, continued for 26 weeks, *n* = 19 Group 2: PSL (as in group 1) followed by maintenance 5–7.5 mg/day for additional 2.5 years, *n* = 30	Relapse	36 months	Relapse rate 61% (group 1) *vs* 24% (group 2), *P* = 0.007
Conventional synthetic DMARDs					
Yunyun *et al.* (2017) [75]	Non-randomized clinical trial	Group 1: PDN 0.5–1.0 mg/kg/day for 1 month then tapered by 5 mg/day to maintenance 5–10 mg/day, *n* = 52 Group 2: PDN (as in group 1) + CYC 50–100 mg/day for 3 months then 50 mg/day or 50 mg every other day, *n* = 50	Relapse rate	12 months	Relapse rate 39% (group 1) *vs* 12% (group 2) Median time to relapse 7 months (group 1) *vs* 11 months (group 2), *P* = 0.018
Yunyun *et al.* (2019) [76]	Open-label RCT	Group 1: PDN 0.6–0.8 mg/kg/day tapered to maintenance ≤10 mg/day, *n* = 35 Group 2: PDN (as in group 1) + MMF 1–1.5 g/day for 6 months then tapered to 0.5–1.0 g/day, *n* = 34	CR, PR	12 months	No statistical difference in CR or PR at 12 weeks Relapses: 40% (group 1) *vs* 21% (group 2), *P* = 0.06
Wang *et al.* (2020) [77]	Open-label RCT	Group 1: PSL 0.5–0.8 mg/kg/day tapered to 10 mg over 6 months, *n* = 33 Group 2: PSL (as in group 1) + LEF 20 mg daily p.o., *n* = 33	Relapse	12 months	Hazard ratio for time to relapse 0.35 (0.13, 0.90, *P* = 0.23), favouring group 2
Biologic DMARDs					
Carruthers *et al.* (2015) [78]	Single-arm open-label trial	RTX 1 g × 2 doses, either monotherapy (*n* = 26) or with concomitant glucocorticoids tapered off over 2 months (*n* = 4)	At 6 months: decline in IgG4-RD RI, no relapses, and no GC use after 2 months	12 months	Primary outcome achieved in 77% 47% in CR at 6 months, 40% at 12 months
Ebbo *et al.* (2017) [79]	Retrospective cohort study	Group 1: no RTX maintenance, *n* = 21 Group 2: RTX administered before relapse (regimens ranging from 300 mg to 1 g, every 1 month to 17 months), *n* = 12	Relapse	Mean 25 months	Mean time to relapse 21 months (group 1) *vs* 41 months (group 2) Hazard ratio for time to relapse 0.10 (0.02, 0.69, *P* = 0.02), favouring group 2
Majumder *et al.* (2018) [80]	Retrospective cohort study (pancreaticobiliary IgG4-RD)	Group 1: RTX induction only (375 mg/m^2^ weekly × 4 or 1000 mg biweekly × 2), *n* = 14 Group 2: RTX induction (as in group 1) and maintenance (375 mg/m^2^ or 1 g every 2–6 months), *n* = 29	Relapse rate	Median 34 months (group 1), 27 months (group 2)	86% CR or PR and off GCs at 6 months after induction Relapse rate 45% (group 1) *vs* 11% (group 2), *P* = 0.034
Campochiaro *et al.* (2020) [81]	Retrospective cohort study	Group 1: RTX induction only (1 g × 2 separated by 15 days), *n* = 7 Group 2a: RTX induction (as in group 1) + maintenance (1 g × 2 every 6 months), *n* = 4 Group 2b: RTX induction (as in group 1) + maintenance (1 g × 1 every 6 months), *n* = 3	Relapse at 18 months	Median 26 months (group 1), 19 months (group 2a), 21 months (group 2b)	71% relapse (group 1) *vs* 0% relapse (group 2), *P* = 0.006
Matza *et al.* (2022) [82]	Single-arm open-label trial	Abatacept 125 mg s.c. weekly, *n* = 10	CR	6 months	CR at 12 weeks in 30% 60% PR at 12 weeks, 50% at week 24
Perugino *et al.* (2023) [83]	Single-arm, open-label trial	Obexelimab 5 mg/kg i.v. every 2 weeks with GCs discontinued within 2 months, *n* = 15	Decline in IgG4-RD RI	6 months	80% met primary endpoint, 93% with any response Median time to response 15 days
Other					
Zhang *et al.* (2019) [84]	Single-arm open-label trial (mild disease)	One i.m. injection of 5 mg betamethasone dipropionate with 2 mg betamethasone sodium phosphate + iguratimod 25 mg p.o. twice daily, *n* = 30	CR, PR	6 months	Week 12: 33% CR, 53% PR Week 24: 30% CR, 57% PR

a Studies specific to AIP were limited to randomized controlled trials only.

AIP: autoimmune pancreatitis; CR: complete remission/response; GC: glucocorticoid; PDN: prednisone; PR: partial remission/response; PSL: prednisolone; RCT: randomized controlled trial; RI: responder index; RTX: rituximab; w/d: withdrawal.

Biologic DMARDs have also been studied in IgG4-RD (Table 2). Rituximab is an anti-CD20 mAb that depletes peripheral B cells. The clinical response to rituximab (and its biosimilar) in patients with IgG4-RD is often swift, leading to significant improvement in disease activity, as observed in two prospective, open-label single-arm trials. Patients are typically treated with 1 g twice over 14 days, and many patients achieve remission with no concomitant oral CS course. Although we frequently use rituximab, it has not been compared with CSs for remission induction, and the optimal dosing and use of concomitant glucocorticoids is unknown. Given the experience with rituximab, other B cell-targeted therapies are being investigated for IgG4-RD (Table 3).

**Table 3.** Ongoing clinical trials in IgG4-related disease

Trial	Design	Study arms	Primary outcome	Follow-up time	Results/status
Conventional synthetic DMARDs					
NCT05746689	Open-label, single-arm trial	Sirolimus + PDN taper	Relapse rate	3 months	Pre-enrolment
Biologic DMARDs					
NCT04918147	Part 1: open-label, single-arm trial Part 2: placebo-controlled RCT	Part 1a/1b: elotuzumab (various regimens) + PDN taper Part 2: elotuzumab + PDN taper *vs* placebo + PDN taper	Part 1: adverse events Part 2: change in IgG4-RD RI	Part 1: up to 48 weeks Part 2: 48 weeks	Part 1 b enrolling
NCT05662241	Placebo-controlled RCT	Obexelimab + PDN taper *vs* placebo + PDN taper	Time to relapse	12 months	Enrolling
NCT04660565	RCT	Belimumab + GC *vs* GC monotherapy	Relapse rate	12 months	Enrolling
NCT05728684	Open-label single-arm trial	CM310 (anti-IL-4 receptor-α mAb)	Response rate	3 months	Pre-enrolment
NCT04540497	Placebo-controlled RCT	i.v. inebilizumab or placebo followed by optional 3-year open-label treatment period	Time to relapse	12 months	Active, no longer enrolling
NCT02705638	Open-label single-arm trial	Rituximab + lenalidomide	Remission	24 months	Completed
Targeted synthetic DMARDs					
NCT05625581	Non-randomized controlled trial	Tofacitinib + GC taper *vs* CYC + GC taper	Remission	6 months	Enrolling
NCT04602598	Open-label single-arm trial	Zanubrutinib	Submandibular and lacrimal gland volume	6 months	Enrolling
NCT04520451	Open-label two-arm trial	Group 1: rilzabrutinib + 12 week GC taper Group 2: placebo + 12 week GC taper followed by crossover to rilzabrutinib	Relapse	12 months	Enrolling
NCT05781516	RCT	Baricitinib + GC taper *vs* GC taper monotherapy	Relapse	12 months	Enrolling

GC: glucocorticoid; IgG4-RD RI: IgG4-related disease responder index; PDN: prednisone; RCT: randomized controlled trial.

T cell-targeted therapies are increasingly being studied in IgG4-RD. Abatacept, an inhibitor of T cell co-stimulation and activation, has been investigated but did not show promising results [82]. Several recent reports [87–89] have noted benefit in patients treated with dupilumab, a monoclonal anti-IL-4 receptor-α antibody, although this has not been studied in a prospective trial.

As of 2023, our usual practice is to induce remission using rituximab. Short course of glucocorticoids (up to 2–3 months) can be used for patients with severe or urgent disease (e.g. cholangitis, aortitis or vision-threatening orbital disease) in whom treatment is needed to prevent irreversible damage.

### Approach to managing patients in remission

Some patients can benefit from maintenance therapy, although this remains poorly studied. First, patients with organ- and life-threatening manifestations (e.g. renal involvement with chronic kidney disease, pancreatic disease with insufficiency) can benefit from maintenance therapy given the risks imposed by disease flare. Second, patients with disease that can be detected only by imaging and in whom routine imaging might be difficult to obtain might benefit. Third, patients with multi-organ disease, elevated baseline serum IgG4 and/or IgE and/or peripheral eosinophilia are at the highest risk for relapse and might benefit from maintenance therapy [19, 90]. The risk of relapse can also vary based on the induction regimen; we routinely find that some patients, even with risk factors for relapse, have quiescent disease for ≥1 year after rituximab. Thus, the approach to maintain remission should be individualized to the specific manifestations of the patient, history of damage, co-morbidities and other factors.

Several maintenance regimens are used. In Asia, it is common to maintain remission with low-dose glucocorticoids (<10 mg/day of prednisone). In patients with IgG4-related pancreatitis, studies have shown a reduced risk of relapse when low-dose glucocorticoids are used compared with observation alone [71, 74], but relapse can occur even on low-dose glucocorticoids. CS-sparing medications have also been evaluated as maintenance therapies. A meta-analysis of 15 studies that included 1169 patients found a lower rate of relapse with a CS-sparing agent in combination with glucocorticoids compared with glucocorticoid monotherapy (odds ratio 0.39, 95% CI 0.20, 0.80) [91]. We and others frequently use rituximab to maintain remission, but this remains poorly studied [91]. When used as maintenance, 1 g of rituximab every 6 months is often used, but this can be spaced further apart depending on the individual patient history. Prospective studies to determine the optimal maintenance regimen are needed.

## Monitoring disease activity and assessing damage in IgG4-RD

Close monitoring of disease activity is important to confirm successful induction of remission and to assess for disease relapses that require retreatment.

### Biomarkers of disease activity and predictors of relapse

Laboratory tests used to monitor disease activity are often the same as those used to establish the diagnosis: serum IgG4 and IgE concentrations, complement levels (C3 and C4) and peripheral eosinophil counts (Table 4). The IgG4 concentration is most frequently used, particularly if it was previously elevated. When elevated at baseline, the IgG4 concentration typically decreases after the initiation of treatment with glucocorticoids, B cell depletion and other therapies [73, 78, 92, 93]. The serum IgG4 might not normalize and can remain elevated even in the absence of disease activity [78, 92, 93]. A rising level can indicate a brewing flare, but when or whether a flare will occur is not always clear [20, 92–96]. In those with hypocomplementaemia, elevated IgE concentrations or peripheral eosinophilia at baseline, recurrent abnormalities can herald a flare. Additionally, some baseline features can identify patients at high risk of relapse, including higher serum IgG4 concentrations, a greater extent of organ involvement, the presence of atopic features, peripheral eosinophilia and elevated serum IgE concentrations [19, 96–98]. Other organ-specific markers (e.g. urinary protein in tubulointerstitial nephritis, bilirubin and alkaline phosphatase in cholangitis) can be used to gauge disease activity at those sites.

**Table 4.** Biomarkers for monitoring IgG4-related disease

Test	Change may herald an IgG4-related disease flare
General laboratory tests	
Immunoglobulin G4	↑
Immunoglobulin E	↑
Eosinophil count	↑
Complement components 3 and 4	↓
ESR	↑
Plasmablast count	↑
Memory B cell count	↑
Organ-specific laboratory tests	
Lipase	↑
Alanine transaminase, aspartate transaminase	↑
Alkaline phosphatase, gamma-glutamyl transferase, bilirubin	↑
Creatinine	↑
Total urine protein to creatinine ratio	↑

### Monitoring disease activity

In patients with disease that is detectable by history and physical examination, this assessment, in combination with a laboratory evaluation, can be adequate to monitor disease activity. Many patients, however, have disease that is more apparent on imaging than on examination; in these cases, serial imaging is often necessary, and the preferred modality will vary by local practice and the organ affected. Disease relapses often affect previously involved organs, but new manifestations can occur, and clinicians should monitor for signs or symptoms thereof. Therefore, disease activity should be reassessed routinely every 3–6 months early on, even in the absence of symptoms, given the frequency of asymptomatic disease in IgG4-RD.

The management of serological relapse (e.g. rising IgG4 concentration) in the absence of a change in manifestations needs to be personalized. Patients with prior involvement of organs that are prone to damage and difficult to assess for active disease (e.g. pancreas) might benefit from treatment, even in the absence of other features of disease activity. In other cases, closer monitoring after a serological relapse is warranted to detect a flare early in its course.

### Damage in IgG4-RD

IgG4-RD can cause irreversible organ damage because of untreated inflammation, the effect of a mass on neighbouring structures or iatrogenic damage related to diagnostic evaluation or treatment. In one series, 58% of patients had damage in at least one organ at diagnosis [99]. Examples of damage include sicca syndrome from resection of a salivary gland, proptosis from a fibrotic orbital pseudotumour, anosmia from sinonasal involvement [100–102], large vessel aneurysms or dissections [103, 104], and chronic ureteral obstruction or tubulointerstitial nephritis leading to chronic kidney disease, including end-stage kidney disease [99, 105–107].

The pancreas is among the most frequently damaged organ from IgG4-RD (Supplementary Table S2, available at *Rheumatology Advances in Practice* online); 60% of patients will have exocrine or endocrine damage at the time of diagnosis [15]. This reflects the indolent nature of pancreatic involvement such that patients will not generally exhibit symptoms until significant damage has occurred. In other forms of chronic pancreatitis, the risk of developing both diabetes and exocrine pancreatic insufficiency is high over the course of a patient’s lifetime with the disease. It is estimated that ≤65% of patients with type 1 AIP have diabetes. The majority of diabetes presents even before CS therapy is initiated [108, 109]. Exocrine pancreatic insufficiency with associated weight loss is seen in ≤50% of patients [110]. Patients with chronic pancreatitis are also at increased risk for osteopenia, osteoporosis and major micronutrient deficiencies [111]. All patients should be screened for diabetes with a haemoglobin A_1c_ annually, for exocrine pancreatic insufficiency with a stool faecal elastase-1 and questions targeted at uncovering symptoms of maldigestion or malabsorption and micronutrient deficiencies, including vitamins A, E, D and K, zinc, selenium, iron, folate, vitamin B12 and magnesium at the time of diagnosis, annually and/or if new symptoms develop [112]. Exocrine pancreatic insufficiency should be managed with pancreatic enzyme replacement therapy at a dose of ≥1000 units/kg per meal and 500 units/kg per meal with snacks. Vitamins should be repleted and monitored annually.

### Differentiating damage from active disease

Differentiating between active disease and damage in IgG4-RD can be challenging. Organ-specific markers, such as markers of cholestasis, proteinuria and glomerular filtration rate, almost always improve with treatment, but they frequently remain abnormal. Likewise, radiological findings (e.g. retroperitoneal fibrosis and mass lesions) often exhibit appreciable improvement after treatment, but in some cases a lack of progression can be a sign of effective treatment owing to the degree of damage that accrued before treatment. Two ways to differentiate damage from active disease include serial evaluations, with the expectation that damage will remain stable over time and active disease will worsen with time, and a trial of treatment with glucocorticoids, which would be expected to improve lesions that are attributable to active disease. Clinicians should be aware of any changes in activity that might suggest a possible malignancy developing at the site of prior IgG4-RD. A clue to this might be disease that was responsive to treatment in the past but now worsening despite resumption of previously effective treatment.

## Future directions and knowledge gaps

Since the initial description of the disease that would become known as IgG4-RD in the early 2000s, knowledge of its epidemiology, pathogenesis and management has expanded dramatically (Table 5). Despite these advances, there are several important avenues for future investigation to improve the care of patients with IgG4-RD. First, further elucidating the pathogenesis of IgG4-RD, including the roles of IgG4 and complement, might identify new targets for therapeutics and approaches to management. Second, although there are now at least two phase 3 clinical trials investigating treatments for remission induction in IgG4-RD (Table 3), studies are also needed to define the risks and benefits of alternative strategies for managing the remission phase of the disease. Third, to inform the design of such studies, an improved understanding of the role of conventional and novel biomarkers of disease activity is needed, because these might guide decision-making. Fourth, although tools exist to measure disease activity objectively for research purposes, there are challenges with their implementation, and future efforts to revise these tools or identify new ones are needed. Fifth, many patients with IgG4-RD have highly fibrotic manifestations of the disease that do not improve substantially with our current treatments. The role of anti-fibrotic treatments is poorly understood and an important priority for future studies. Finally, IgG4-RD is a rare disease, and many patients express interest in connecting with other patients with this disease and in learning strategies to manage the uncertainty that comes with having IgG4-RD. At this time, there are no formal education or support resources for patients with IgG4-RD.

**Table 5.** Future directions

Elucidating the pathogenesis of IgG4-related disease Role of IgG4 Significance of complement in pathogenesis and as a potential treatment target Developing an effective animal model of IgG4-RD
Identifying safe and effective CS-sparing treatment strategies Investigating B cell-targeted therapies in randomized controlled trials Exploring the role of anti-fibrotic therapies for patients with highly fibrotic manifestations Comparing alternative strategies for maintenance of remission
Improving our understanding of the role of conventional and novel biomarkers of disease activity Circulating biomarkers like IgG4, complement, eosinophilia and IgE might be useful for guiding management decisions but require further study Novel imaging biomarkers might better classify patients into relevant disease groups with implications for treatment (e.g. might benefit from anti-fibrotic *vs* anti-inflammatory treatments)
Refining instruments to measure disease activity from both the clinician and patient perspective
Identifying ways to support patients through their journey with this IgG4-RD Improving patient-facing resources on this disease Developing support groups for patients Identifying intervention that may improve quality of life and well-being in the face of a rare disease

## Supplementary Material

rkae020_Supplementary_Data

## Data Availability

No new data were generated or analysed in support of this article.
