# Supplementary material for: Current and future advances in practice: IgG4-related disease
Source: Rheumatol Adv Pract. 2024 Apr 10;8(2):rkae020. doi: 10.1093/rap/rkae020 (PMC11003820; doi:10.1093/rap/rkae020)
Supplement: rkae020_Supplementary_Data [file rkae020_supplementary_data.docx]

**Supplementary Material for:** **“Current and future advances in practice: IgG4-related disease”**

Supplementary Table S1: Comparison of Type 1 and Type 2 Autoimmune Pancreatitis (AIP)

| **Clinical Features**  (10, 105, 113-117) | **Type 1 AIP** | **Type 2 AIP** |
| --- | --- | --- |
| **Age (years)** | 61-66 | 34-53 |
| **Male (%)** | 77% | 55% |
| **Initial Symptom** |  |  |
| Jaundice | 70% | 30% |
| Abdominal Pain | 10% | 50% |
| Acute Pancreatitis | <5% | 50% |
| **Imaging Findings** |  |  |
| Diffuse swelling | 40% | 28% |
| Other features | 60% | 72% |
| Sampled diagnostic | 43% | 44% |
| **Elevated IgG4 level**  **(>140 mg/dL)** | 70-80% | ~20% |
| **Other Organ Involvement** | 60% | 0 (excluding IBD) |
| **IBD association** | 6% | 25-44% |
| **Relapse rate** | 47% | <10% |

Supplementary Table S2: Manifestations and Management of Pancreatic Damage

| **Manifestations of Damage** | **Diagnostic Strategy** | **Management** |
| --- | --- | --- |
| **Exocrine Pancreatic Insufficiency** | -        Screen for symptoms of maldigestion: bloating, steatorrhea, nausea, loose stool, post-prandial pain, nausea, vomiting, unintentional weight loss | Pancreatic lipase 1000-1500 Units/kg/meal and 500-750 Units/kg/meal |
|  | -        Stool based testing: fecal elastase, 72 hour fecal fat | Dietary counseling |
|  | -        Screen for presence of micronutrient deficiencies (Vitamin A, E, D, K, zinc, selenium, folate, B12, magnesium) |  |
| **Diabetes** | Fasting glucose or Annual A1c | Consider specialized diabetic care as patients at risk for Type 3C DM |
|  |  | Metformin, Sulfonylurea, Dipeptidyl peptidase-4 inhibitor (DLP-4 inhibitor), SGLT2 inhibitor, |
| **Micronutrient deficiencies** | Annual vitamin screening (Vitamin A, E, D, K, zinc, selenium, folate, B12, magnesium) | Repletion of vitamin deficiencies and repeat blood work in 3 months to ensure correction. |
